# Aspects related to the inference of causality in cross-sectional studies. Comments on: “8-Hydroxy-2’-deoxyguanosine protein immunoexpression is associated with the pathogenesis of actinic cheilitis”^☆^

**DOI:** 10.1016/j.abd.2024.03.002

**Published:** 2024-06-13

**Authors:** Ivanka Miranda de Castro Martins, Anna Carolina Miola, Luiz Eduardo Fabrício de Melo Garbers, Hélio Amante Miot

**Affiliations:** Department of Infectology, Dermatology, Imaging Diagnosis and Radiotherapy, Faculty of Medicine, Universidade Estadual Paulista, Botucatu, SP, Brazil

*Dear Editor,*

Due to the potential for malignancy and contiguity to the photoprotected mucosa, actinic cheilitis (AC) represents the archetype of the activity of the cutaneous field cancerization (CFC), an opportune model for research about carcinogenesis in *anima nobile*.^1^ Therefore, the article by Varela et al., which explored the expression of 8-OHdG in AC, concluding for association with pathogenesis, was read with interest.^2^ The authors should be congratulated; however, some comments related to the generalization of the results are essential.

The search for histopathological markers of CFC activity is relevant for its characterization, understanding of the pathogenesis, and to define outcome in therapeutic prospection. To date, no high-performance markers have been identified; however, the expression of p53, Ki67, detection of cyclobutane-thymidine dimers, and degree of epithelial dysplasia are the most studied ones.^3^, ^4^

While ultraviolet radiation induces oxidative stress in the epithelium and topical antioxidants reduce CFC activity, markers of oxidative damage in DNA are candidates for characterizing CFC activity.^5^ 8-OHdG expression is rapidly increased in sun-exposed epithelium, remaining for days after a radiation dose; however, it can also be induced by other oxidative agents, such as smoking and underlying inflammation.

Human skin is an organ which interacts with the environment and has, during evolution, developed DNA repair, apoptotic, and antioxidant mechanisms. Their failure justifies the higher incidence of neoplasms in syndromes (e.g., xeroderma pigmentosum), immunosuppressed patients, and the elderly. Lower lips are chronically exposed to the sun, justifying the regular expression of markers of oxidative damage. However, this study did not include a control group with healthy, photoexposed lips, nor was the photoprotected mucosa sampled, which would help in the comparative evaluation of 8-OHdG expression patterns.

The lack of association between the 8-OHdG histopathological score and the degree of AC dysplasia or smoking does not corroborate the expectation of a biological gradient. Moreover, the expression of 8-OHdG in AC, although intense, does not allow inferring that it is causally involved in the etiopathogenic process, or that it is a by-product of the epithelium with dysfunctional antioxidant mechanisms.

Finally, the nuclear and cytoplasmic expression of 8-OHdG must be analyzed separately, since damage to mitochondrial DNA induces autophagy, whereas nuclear damage is potentially carcinogenic. Although the authors declared that there was predominantly nuclear expression, but cytoplasmic too, there was no differential analysis, which makes the interpretation of these results difficult.

Therefore, the conclusion about the involvement of 8-OHdG in the pathogenesis of AC should be interpreted with caution.

## Financial support

None declared.

## Authors' contributions

Ivanka Miranda de Castro Martins: Design and planning of the study, drafting and editing of the manuscript; approval of the final version of the manuscript.

Anna Carolina Miola: Design and planning of the study, drafting and editing of the manuscript; approval of the final version of the manuscript.

Luiz Eduardo Fabrício de Melo Garbers: Design and planning of the study, drafting and editing of the manuscript; approval of the final version of the manuscript.

Hélio Amante Miot: Design and planning of the study, drafting and editing of the manuscript; approval of the final version of the manuscript.

## Conflicts of interest

None declared.
